# Ganoderic Acid A Ameliorates Pentylenetetrazol‐Induced Epilepsy in Mice via Activating KCNQ2

**DOI:** 10.1002/cns.71152

**Published:** 2026-09-24

**Authors:** Yang Yao, Zhujun Liu, Xujiao Wang, Cunhao Bian, Rui Wang, Yukun Zhang

**Affiliations:** ^1^ Chongqing Key Laboratory of Development and Utilization of Genuine Medicinal Materials in Three Gorges Reservoir Area Chongqing China; ^2^ Department of Clinical Pharmacology, College of Pharmacy Dalian Medical University Dalian China; ^3^ Department of Pharmacology, College of Pharmacy Dalian Medical University Dalian China; ^4^ State Key Laboratory of Natural and Biomimetic Drugs, Department of Pharmacology, School of Basic Medical Sciences Peking University Beijing China; ^5^ Natural Drug Intervention in Aging Key Laboratory of Chongqing Education Commission Chongqing China

**Keywords:** epilepsy, Ganoderic acid A, hippocampal neuron, KCNQ2, neuroinflammation

## Abstract

**Trial Design:**

This study aimed to explore the antiepileptic effect of Ganoderic acid A (GA‐A) on pentylenetetrazol (PTZ)‐induced epilepsy in mice and clarify its underlying mechanism, focusing on the KCNQ2 channel.

**Methods:**

C57BL/6 mice and hippocampal‐specific KCNQ2 knockout mice were used to establish PTZ‐induced acute epilepsy models. GA‐A was pretreated for 7 days. Epileptic seizures were evaluated by the Racine score and electroencephalogram (EEG). Neuronal injury was detected by HE and TUNEL staining. Neuroinflammation and oxidative stress were assessed by measuring inflammatory factors and oxidative markers. The target interaction between GA‐A and KCNQ2 was verified by protein chip, molecular docking, surface plasmon resonance (SPR), and electrophysiological recording.

**Results:**

GA‐A dose‐dependently reduced seizure severity, shortened epileptiform discharge duration, alleviated hippocampal neuronal loss and apoptosis, and inhibited neuroinflammation and oxidative stress in PTZ‐treated mice. GA‐A directly bound to KCNQ2 and activated KCNQ2/3 channels to generate M current. Hippocampal KCNQ2 knockout weakened the ameliorative effect of GA‐A on EEG abnormalities and epilepsy‐related anxiety‐ and depression‐like behaviors, but did not affect its anti‐inflammatory and antioxidant effects.

**Conclusion:**

GA‐A attenuates PTZ‐induced epilepsy in mice via activating the KCNQ2 channel to suppress neuronal hyperexcitability and independently inhibiting neuroinflammation and oxidative stress. GA‐A is a promising KCNQ2 activator and lead compound for antiepileptic drug development.

## Graphical abstract

GA‐A binds and activates KCNQ2, thereby reducing epileptic‐like EEG discharges, the loss and apoptosis of hippocampal neurons, and improving anxiety and depression‐like behavioral changes. In addition, GA‐A inhibits neuroinflammation and alleviates oxidative stress independent of activating KCNQ2.

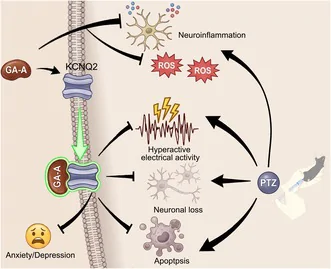

## 1 Introduction

Epilepsy is a common primary episodic chronic disease of the nervous system, leading to short‐term functional impairment of the brain [1]. At present, the treatment of epilepsy mainly relies on drug therapy. Traditional antiepileptic drugs include phenobarbital, phenytoin, sodium valproate, etc., but these drugs can cause side effects such as drowsiness, dizziness, nausea, and vomiting [2]. However, about one‐third of patients with intractable epilepsy cannot have their condition effectively controlled by traditional drugs. The recurrence of epilepsy and long‐term medication has brought a heavy burden to patients, their families, and society. Therefore, there is an urgent need for new antiepileptic drugs with definite efficacy, few side effects, and good tolerance.

Many types of epilepsy are often related to abnormal ion channel function [3]. Ion channel dysfunction leads to abnormal neuronal excitability. If the abnormal neuronal excitability cannot be inhibited, then epilepsy occurs [4]. Clinically, antiepileptic drugs acting on ion channels are commonly used to treat epilepsy. Potassium channel opener KCNQ2/3 voltage‐gated potassium channel activators are one of the choices for drug‐resistant epilepsy [5]. Retigabine (RTG) is the first KCNQ2/3 channel activator to be discovered, and in 2011 it was approved for external use as an adjunctive drug for drug‐resistant epilepsy, with good therapeutic efficacy. However, long‐term use can lead to side effects such as retinal pigmentation and reduced visual acuity [6]. Although there have been many reports on KCNQ2 channel activators since 2017, the unclear molecular mechanism has hindered further development, and the binding pocket of KCNQ2 for small molecules remains unclear. With the recent elucidation of the crystal structure of the KCNQ2/3 channel protein, it has greatly promoted research on the binding sites of small molecules with KCNQ2/3 and the key amino acid residue sites. Taking KCNQ2/3 as the target, research on the binding sites of small molecules and the key amino acid residue sites will greatly promote the development of new antiepileptic drugs.

*Ganoderma lucidum* ( *G. lucidum* ), a renowned traditional Chinese fungal medicine first documented in Shennong's Herbal Classic, refers to the dried fruiting bodies of *G. lucidum (Leyss. ex Fr.) Karst*. belonging to the Polyporaceae family. For centuries, it has been valued for its effects of replenishing qi, calming the spirit, relieving coughs, and alleviating asthma. Modern phytochemical studies have identified triterpenoids, especially Ganoderic acids, as the major bioactive components, among which Ganoderic acid A (GA‐A) has emerged as a research focus due to its diverse pharmacological activities [7]. GA‐A is a highly oxidized lanostane‐type tetracyclic triterpenoid, and it exhibits prominent anti‐inflammatory, anti‐tumor, lipid‐regulating, and organ‐protective properties [8, 9]. Previous studies have indicated that *G. lucidum* extract may improve central and peripheral nervous system diseases by nourishing nerves [10], inhibiting neuroinflammation, and reducing oxidative stress [11]. *G. lucidum* reduces neuroinflammation and oxidative stress in the brain, thereby protecting neurons from damage and improving cognitive function in animal models of Alzheimer's disease [12, 13, 14]. *G. lucidum* extract also has an improving effect on other age‐related neurodegenerative diseases [15]. Moreover, *G. lucidum* polysaccharides may inhibit calcium overload and promote CaMKIIα expression, thereby protecting epileptic neurons [16]. *G. lucidum* spores may protect hippocampal neurons by promoting neurotrophin‐4 expression and inhibiting N‐Cadherin expression [17]. The previous studies point out that GA‐A exhibits strong antiepileptic effects by reducing cortical neuron apoptosis and expression of calcium‐sensing receptors, stimulating the MAPK pathway [18], alleviating neuroinflammation [19], and enhancing the antioxidant activity of the mitochondrial membrane [20]. However, its direct target and potential mechanism are still unclear.

The importance of natural products in treating human diseases has been fully proven. They usually have definite efficacy and few adverse reactions, but their application is often limited because the targets are not clear. In this study, it was found that GA‐A could activate the KCNQ2/3 channel and produce M current. In animal experiments, GA‐A could alleviate epilepsy induced by pentylenetetrazol (PTZ), including inhibiting neuroinflammation, changes in brain electrical activity, loss of neurons, and ameliorating PTZ‐induced anxiety‐ and depression‐like behavior. Therefore, it is proposed that GA‐A activates the KCNQ2/3 channel, which can inhibit the excessive excitability of neurons and synaptic weight loss, thus playing an “antiepileptic” role. By using hippocampal‐specific KCNQ2 knockout mice, we found that GA‐A ameliorates PTZ‐induced changes in brain electrical activity and anxiety‐ and depression‐like behavior that depend on activating KCNQ2 but inhibit neuronal inflammation and oxidative stress independent of KCNQ2. This project clarified the antiepileptic effect and the direct target of GA‐A, which is of great significance for the research of antiepileptic drugs. The successful development of GA‐A as a drug is expected not only to promote the development of GA‐A into a drug but also to promote research on the molecular mechanism of new KCNQ2/3 activators.

## 2 Materials and Methods

### 2.1 Drugs

GA‐A was purchased from Chengdu Pfeiffer Biotechnology Co. Ltd. (Chengdu, China). The serum biochemical detection kit was purchased from Nanjing Jiancheng Bioengineering Co. Ltd. Unless otherwise specified, all other reagents were purchased from Sigma (Sigma‐Aldrich, St. Louis, MO, USA).

### 2.2 Animals

Adult male C57BL/6 mice (19–23 g) were employed in this study. They were prepared under SPF conditions at Chongqing Three Gorges Medical College. The mice were maintained in the animal house at a temperature of 25°C and a light/dark cycle of 12/12 h with normal diet. Groups of eight mice were chosen randomly for the studies. In all tests on animals, they were euthanized by excessive anesthesia after completing the experiments.

### 2.3 Seizure Induction by PTZ

Acute epileptic seizures were induced in mice by administering 50 mg/kg of PTZ [21]. Seizures were scored according to Racine's criteria [22]. Seizure stages were classified as follows: Stage 0, no response; Stage 1, ear and facial twitching; Stage 2, myoclonic jerks; Stage 3, clonic forelimb convulsions; Stage 4, generalized clonic seizures with turning to a side position; and Stage 5, generalized tonic–clonic seizures or death. Mice with at least three consecutive seizures of Stage 4 or 5 were defined as fully kindled.

### 2.4 Drug Administration

Animals were divided into five groups, and each group had eight animals: the control group, the PTZ group, and the PTZ + GA‐A (5, 10, 20 mg/kg) group. The dosage of intraperitoneal injection of GA‐A was administered according to the literature [23]. GA‐A was administered continuously for 7 days. Control and PTZ groups were administered the same amount of vehicle by intraperitoneal injection.

### 2.5 Electroencephalography (EEG) Measurement

After PTZ injection, two bipolar tungsten electrodes (Cat No. 796000, A‐M Systems) and a bipolar stainless‐steel electrode were respectively implanted into the bilateral ANT and the CA1 region of the right hippocampus of the mouse. EEG data were collected and analyzed using the NicoletOne EEG System (parameters: 1–70 Hz low‐ and high‐frequency filters, 15 mm/s recording speed; Natus). According to the Racine scale, spontaneous recurrent seizures on a scale of 4~5 and their duration were observed and recorded.

### 2.6 Open Field Test (OFT)

OFT was performed according to a previously published article with some modifications [24]. Briefly, mice were individually placed in an opaque, non‐reflective open‐field arena (40 cm × 40 cm × 40 cm) for a 10 min free exploration. After each test, the arena was thoroughly cleaned with 70% ethanol and air‐dried to eliminate residual olfactory cues. An overhead infrared camera continuously monitored the locomotor activity and movement trajectories of mice for full behavioral detail capture. Behavioral data were analyzed offline via a professional video tracking system, with two key parameters recorded: total travel distance (an indicator of locomotor activity) and center zone residence time. The center zone was defined as a 20 × 20 cm central square, and a marked reduction in its residence time was indicative of increased anxiety‐like behavior in mice.

### 2.7 Novel Object Recognition (NOR) Test

For the NOR test, there were three phases. For the adaptation phase, mice were housed in an empty chamber for 10 min daily over 3 consecutive days. On day 4, the familiarization phase was performed: mice were first acclimated to the empty chamber for 2 min, then allowed 10 min of free exploration with two identical, non‐irritating cylindrical plastic objects (5 cm in height, 3 cm in diameter) placed symmetrically in the chamber. A 10 min interphase delay followed, during which mice were returned to their home cages. In the subsequent testing phase, one familiar object was replaced with a novel one (distinct in shape, matched in size and texture), and mice were given 10 min of free exploration in the chamber with one familiar and one novel object. After each phase for every mouse, the chamber interior and all objects were thoroughly cleaned with 70% ethanol and air‐dried to eliminate olfactory and tactile cue interference. The recognition index was calculated as follows: recognition index % = (time spent with the novel object/total time spent with both objects) × 100%.

### 2.8 Forced Swim Test (FST)

The forced swimming test, also known as the behavioral despair test, is used to study depression in rodents [25]. Mice were given a standard bucket (40 cm high and 28 cm in diameter) to swim in water for 6 min (water temperature was 25°C), ensuring that their lower limbs did not touch the bottom of the bucket. The latency to immobility, the total duration of active swimming, and the time spent immobile were recorded during the 6 min period in which the animals were in the container.

### 2.9 Tail Suspension Test (TST)

Mice were suspended on a 55 cm high laboratory rack by adhesive tape positioned about 1 cm from the tail tip. The approximate distance between the animal's nose and the operating floor was 20–25 cm. Record the cumulative immobility time of the animals during the 6‐min suspension period.

### 2.10 Hematoxylin and Eosin (HE) Staining

The entire brain samples were fixed with 4% paraformaldehyde and dehydrated. They were embedded in paraffin and cut into 4 μm sections. Then the samples were stained with hematoxylin and eosin (Beyotime Biotechnology, Jiangsu, China). The slices were observed under an optical microscope.

### 2.11 Enzyme‐Linked Immunosorbent Assay (ELISA)

Tumor necrosis factor (TNF)‐α, interleukin (IL)‐1β, IL‐6, interferon (IFN)‐γ, nitric oxide (NO), and glial fibrillary acidic protein (GFAP) were measured using a commercial ELISA test kit (Jiangsu Meibiao Biological Technology Co. Ltd., Jiangsu, China) according to the manufacturer's instructions.

### 2.12 Detection of Biochemical Indicators

According to the manufacturer's instructions, the levels of malondialdehyde (MDA) were measured using a colorimetric assay kit (Nanjing Jiancheng Bioengineering, Nanjing, China).

### 2.13 Immunohistochemistry

For immunostaining, paraffin tissue sections were incubated in 3% hydrogen peroxide solution to inactivate endogenous peroxidases. Antigen retrieval was performed by heating the sections in citrate buffer (pH 6.0). Following serum blocking, the sections were incubated with diluted KCNQ2 Monoclonal antibody (1:100, 66774‐1‐Ig, Proteintech, Wuhan, China) or NeuN monoclonal antibody (1:50, 66836‐1‐Ig, Proteintech) and subsequently with HRP‐conjugated secondary antibodies (1:100, Proteintech). Immunohistochemical staining was visualized using a DAB chromogenic kit (Beijing Solarbio Science & Technology Co. Ltd., Beijing, China).

### 2.14 Electrophysiological Characterization in Xenopus Oocytes

Xenopus oocytes were surgically isolated from *Xenopus laevis* . Stage V–VII oocytes were defolliculated with 2 mg·mL^−1^ collagenase Type I (Sigma‐Aldrich, St. Louis, MO, USA) in Ca^2+^‐free saline (96 mM NaCl, 2 mM KCl, 5 mM MgCl_2_, 5 mM HEPES, pH 7.6) for 0.5–1 h at room temperature. Oocytes were co‐injected with 23 nL sterile water containing 10 ng RNA encoding wild‐type *Kcnq2*, 10 ng RNA encoding *Kcnq3*, or 23 nL sterile water alone.

Whole‐cell patch‐clamp recordings were performed at room temperature (22°C–24°C) to record M‐type potassium currents (M‐currents) in *Xenopus laevis* oocytes with heterologous channel expression. Borosilicate glass patch pipettes with a tip resistance of 1.0–1.5 MΩ were used for recordings. The extracellular bath solution contained 96 mM NaCl, 2 mM KCl, 1.8 mM CaCl_2_, 1 mM MgCl_2_, 5 mM HEPES, pH 7.4. The intracellular pipette solution consisted of 120 mM K‐gluconate, 5 mM KCl, 2 mM MgCl_2_, 10 mM HEPES, pH 7.2. Solutions were freshly prepared and filtered before use. The oocyte vitelline membrane was manually removed before recording to enable gigaseal formation. Under microscopic guidance, gentle suction was applied to form a stable gigaseal (≥ 2 GΩ), and brief suction pulses were used to establish the whole‐cell configuration. Series resistance was compensated at 70%–80%, and capacitive transients were automatically corrected via the amplifier system. In this study, a total of *N* = 10 valid oocytes were recorded for each experimental group. All oocytes were obtained from more than 3 individual animals as independent biological replicates. The experiments were performed in 3 independent experimental batches on different days to eliminate batch variation. Only oocytes with stable sealing and minimal current rundown were included for data analysis. For channel activation, oocytes were held at −90 mV and depolarized in 10 mV steps from −80 to +80 mV for 500 ms every 5 s. Wild‐type currents were recorded in parallel with mutant channels from the same oocyte batch to control for expression variability. Currents were recorded before drug application, during drug exposure, and after washout. Peak currents were measured at the end of each test pulse. Statistical analysis was performed using GraphPad Prism 8.

### 2.15 DNA Isolation and Gene Type

DNA was extracted from the tail tissue, and PCR was performed using the direct PCR Kit (Bioman (Beijing) Technology Co. Ltd., Beijing, China). For the identification of *Kcnq2* flox alleles, primer pair F2 (5′‐TATACGGAAGGAGAAATGAAAACCG‐3′) and R2 (5′‐TGCAACAATTCCTTCCTAGGTTCA‐3′) was used, yielding a 200 bp band for *Kcnq2* allele and a 131 bp band for the wild‐type (WT) allele; *Kcnq2* flox/flox homozygotes showed a single 200 bp band, *Kcnq2* flox/+ heterozygotes showed both 200 and 131 bp bands, and WT mice showed a single 131 bp band. For Cre recombinase genotyping, universal Cre primers F (5′‐CATATTGGCAGAACGAAAACGC‐3′) and R (5′‐CCTGTTTCACTATCCAGGTTACGG‐3′) were used, with a 413 bp band indicating Cre‐positive mice. For the verification of *Kcnq2* gene deletion in target tissues, primer pair F2 and R1 (5′‐CTTGGGAATGGAAGGCCAAGATA‐3′) was applied, where a 264 bp band indicated successful gene deletion, and a 200 bp band indicated no deletion despite Cre expression. Negative control (WT mouse genomic DNA) and blank control (ddH_2_O) were included in all PCR reactions to exclude non‐specific amplification.

### 2.16 Western Blot Analysis

For the Western blot assay, total proteins were extracted from the hippocampus tissue using the whole protein extraction kit (Keygen Biotech, Nanjing, China). Protein concentration was determined by the BCA Protein Assay Kit (Bioman Beijing Technology Co. Ltd., Beijing, China). Then, equal amounts of total proteins (20 μg per lane) were separated by 10% sodium dodecyl sulfate‐polyacrylamide gel electrophoresis (SDS‐PAGE) and transferred to polyvinylidene fluoride (Amersham, Buckinghamshire, UK) membranes. The membranes were blocked in 5% non‐fat milk and incubated with antibodies against KCNQ2 and GAPDH (Cell Signaling Technology, MA, USA) diluted at 1:1000. GAPDH was used as an internal standard. Specific proteins were detected with the qTouch Western Blot Imager (RWD Life Science Co. Ltd., Shenzhen, China). The protein bands were quantified using the Quantity One software.

### 2.17 TdT‐Mediated dUTP Nick‐End Labeling (TUNEL) Staining

The TUNEL staining kit was purchased from Elabscience Biotechnology Co Ltd. TUNEL staining was performed as indicated previously [26]. Briefly, tissues were fixed with 4% paraformaldehyde for 20 min and then permeabilized with 0.1% Triton X‐100 in PBS for 5 min at room temperature. Then, samples were incubated with the FITC‐conjugated TUNEL reaction mixture in a humidified dark chamber at 37°C for 60 min. Nuclear counterstaining was performed with DAPI for 5 min. Fluorescence microscopy observation was conducted. Apoptotic cells exhibited green FITC signals, whereas all nuclei displayed blue DAPI fluorescence.

### 2.18 Huprot Human Protein Assay

The human protein microarray assay, which contains 20,000 proteins, and data analysis were conducted by Arrayit HuProtTM v2.0 19K Human Proteome Microarrays (CDI Laboratories, Baltimore, MD), according to the previously published procedure [23]. The signal‐to‐noise ratio (SNR) was calculated as the ratio of the foreground value to the background value. For each protein dot, a SA‐Cy3 group signal vs. Bio‐SA‐Cy3 signal of ≥ 1.5 was identified as a positive result.

### 2.19 Molecular Docking

Molecular docking was performed to predict the binding mode between GA‐A and KCNQ2. A semi‐flexible docking strategy was adopted to simulate the formation of stable complexes, which is essential for elucidating mechanisms of action and for the identification of potential lead compounds. Docking calculations were conducted using AutoDock Vina version 1.2.7, where GA‐A (PubChem CID: 471002) was docked to KCNQ2 A‐D chains (PDB ID: 7CR0). Protein structures were preprocessed with PyMOL version 2.4 by removing water molecules and non‐essential ligands, followed by the addition of hydrogen atoms. The ligand structure was energy‐minimized using ChemDraw version 20.0. PDBQT files were generated with AutoDock Tools version 1.5.6, and the docking grid box was defined to cover the entire protein structure, while all other parameters were kept at default values.

### 2.20 Surface Plasmon Resonance (SPR)

Biacore T200 Protein Interaction Assay system (GE Healthcare) with a CM5 sensor chip was used for the experiment. Before immobilization on a sensor chip, the KCNQ2 protein was diluted to a final concentration of 10 μM in sodium acetate. It was also injected through the channel. Then 1 M ethanolamine, pH 8.5, was injected over the channels to cap the chip's surface. KCNQ2 binding affinity with GA‐A was determined by injecting different doses of GA‐A into KCNQ2‐immobilized chambers (1–200 nM). Results were analyzed using Biacore T200 Manager software. Binding kinetic parameters were calculated by global fitting of the kinetic data from various concentrations of GA‐A using a 1:1 Langmuir binding model.

### 2.21 Statistical Analysis

All experiments are randomized and blinded. Data from more than 3 independent experiments are expressed as Mean ± SD. The exact group size (*n*) for each experimental group/condition is provided. Statistical analysis was performed with GraphPad Prism 8.0 software (San Diego, CA, USA). Student's *t*‐test was used to compare two groups, and one‐way ANOVA or the non‐parametric Kruskal–Wallis test, followed by Dunn's post hoc test, was used to compare three or more independent groups. *p* values of < 0.05 were considered statistically significant.

## 3 Results

### 3.1 GA‐A Reduces the Frequency and Duration of Epileptic Seizures, Inhibits Oxidative Stress and Inflammation Caused by PTZ

**FIGURE 1:**
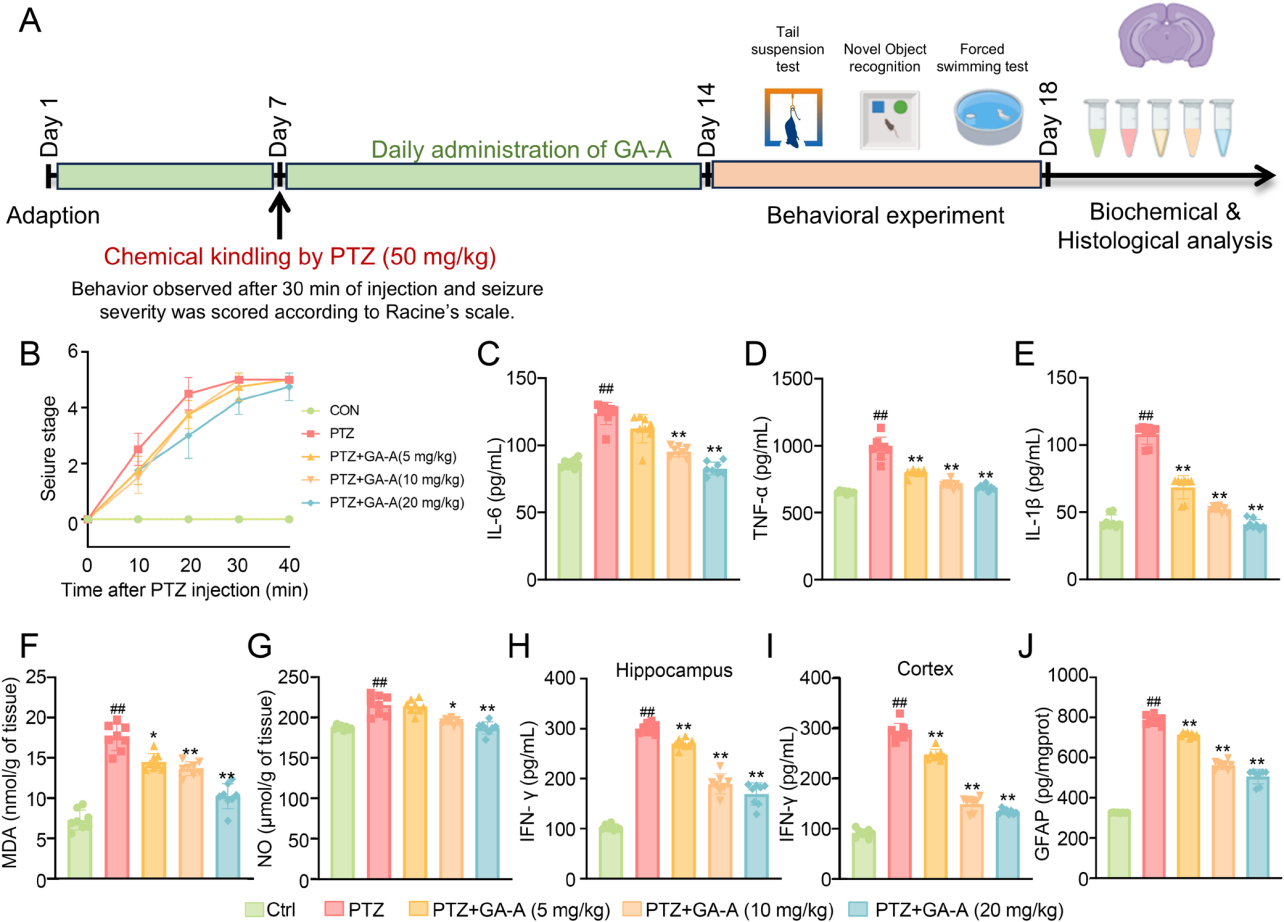
GA‐A alleviates epilepsy via mitigation of neuroinflammation and astrogliosis. (A) Experimental schedule. (B) Racine's scale. (C) Serum IL‐6 level. (D) Serum TNF‐α level. (E) Serum IL‐1β level. (F) Hippocampus MDA level. (G) Hippocampus NO level. (H) Hippocampus IFN‐γ level. (I) Cortex IFN‐γ level. (J) Hippocampus GFAP expression. Data were shown as mean ± SD, *n* = 8, ^##^ *p* < 0.01 compared with the Ctrl group; *p < 0.05, **p < 0.01 compared with the PTZ group.

Chemical kindling was induced by intraperitoneal injection of PTZ (50 mg/kg) following 7 days of daily pre‐administration with GA‐A (5, 10, or 20 mg/kg) or vehicle (Figure 1A). Seizure severity was scored according to Racine's scale. As shown in Figure 1B, PTZ administration resulted in a high seizure stage. At the same time, GA‐A treatment, particularly at 20 mg/kg, significantly reduced the seizure severity compared to the PTZ group. The time‐course analysis of seizure progression (Figure 1B) revealed that GA‐A dose‐dependently delayed and reduced the onset and progression of seizures induced by PTZ, with the 20 mg/kg dose showing the most pronounced effect. To assess the anti‐inflammatory effects of GA‐A, serum IL‐6, TNF‐α, and IL‐1β were detected. As depicted in Figure 1C–E, PTZ treatment significantly increased the levels of IL‐6, TNF‐α, and IL‐1β compared to the control (Ctrl) group. In contrast, GA‐A administration (5, 10, and 20 mg/kg) dose‐dependently attenuated these elevations. Oxidative stress is a key contributor to seizure‐induced brain damage. We measured the levels of MDA, a marker of lipid peroxidation, and NO in the hippocampus. PTZ administration significantly increased MDA (Figure 1F) and NO (Figure 1G) levels compared to the Ctrl group. GA‐A treatment (10 and 20 mg/kg) significantly reversed these increases, indicating a reduction in oxidative stress. We next evaluated the levels of IFN‐γ in the hippocampus and cortex, as well as the expression of GFAP, a marker of astrogliosis [27]. PTZ treatment significantly elevated IFN‐γ levels in both the hippocampus (Figure 1H) and cortex (Figure 1I), as well as GFAP expression (Figure 1J). GA‐A administration significantly reduced IFN‐γ levels and GFAP expression in a dose‐dependent manner. The above results suggest GA‐A alleviates epilepsy via mitigation of neuroinflammation and astrogliosis.

### 3.2 GA‐A Alleviates Neuronal Loss and Cell Apoptosis Caused by PTZ

**FIGURE 2:**
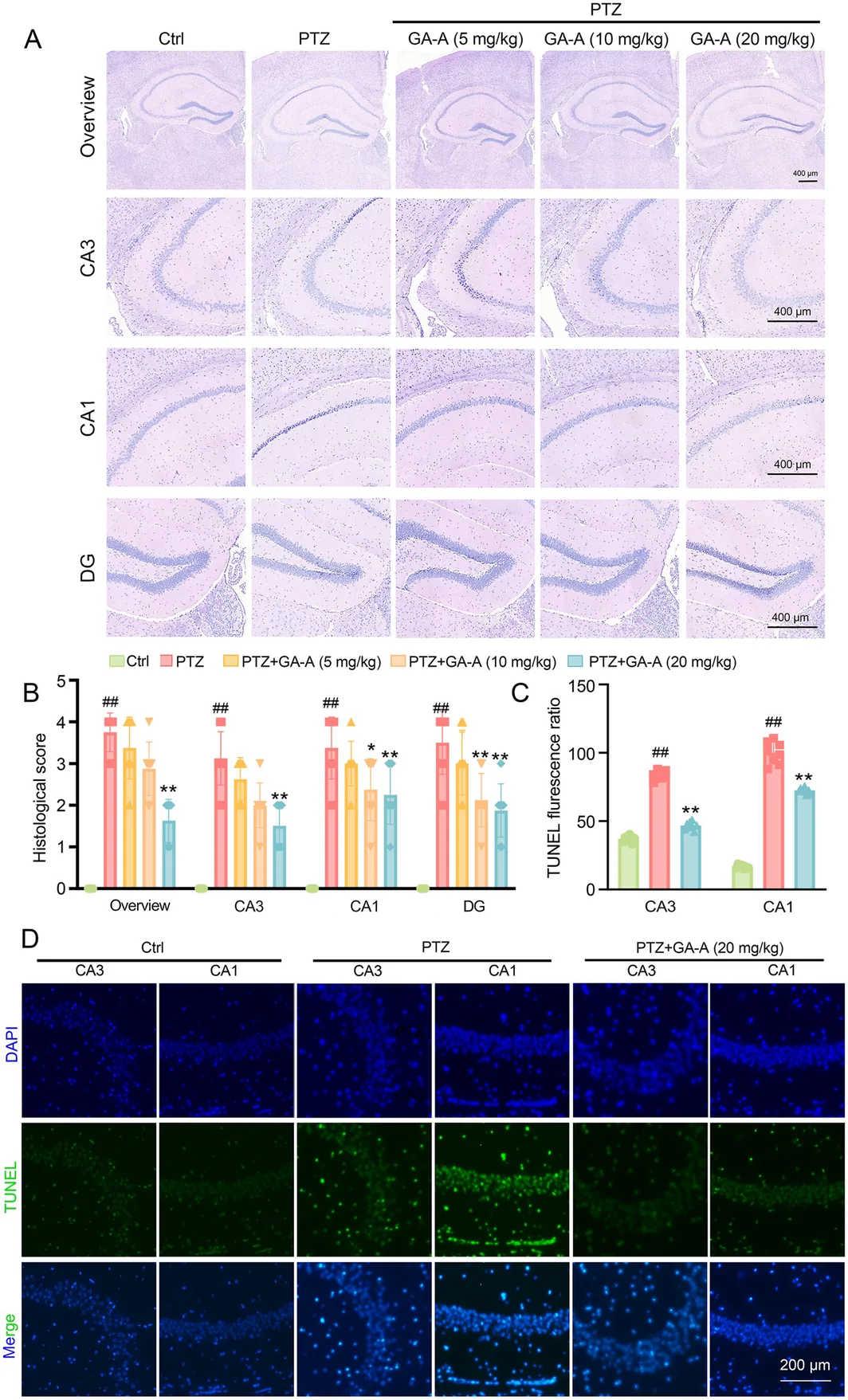
GA‐A alleviates neuronal loss and cell apoptosis caused by PTZ. (A) HE staining of the hippocampus. Scale bar = 400 μm. (B) Histological score. (C) TUNEL fluorescence ratio. (D) Representative photos of TUNEL staining. Scale bar = 200 μm. Data were shown as mean ± SD, *n* = 8, ^##^ *p* < 0.01 compared with the Ctrl group; *p < 0.05, **p < 0.01 compared with the PTZ group.

HE staining showed that the Ctrl group had intact hippocampal structure, neatly arranged vertebral neurons, intact cell bodies, clear nucleoli, and no atrophy or vacuolization. The granular cells in the DG form a dense, well‐defined layer, with no significant pathological changes. The cell layer of the vertebral body in the model group is disorganized, with pronounced neuronal loss, nuclear condensation, and cytoplasmic vacuolization. Inflammatory cell infiltration is present in the surrounding parenchyma. The low‐dose group showed a mild improvement relative to the model group, whereas the high‐dose group showed hippocampal morphology comparable to control levels. The present results showed a dose‐dependent protective effect of GA‐A on neuronal damage (Figure 2A,B). Moreover, TUNEL staining showed that GA‐A significantly reduced apoptotic cells in the CA1 and CA3 regions of the hippocampus (Figure 2C,D). The results indicated that GA‐A can relieve PTZ‐induced neuronal damage and cell apoptosis.

### 3.3 GA‐A Alleviates PTZ‐Induced Epileptic Seizures, Improves Anxiety and Depression Like Behavior

**FIGURE 3:**
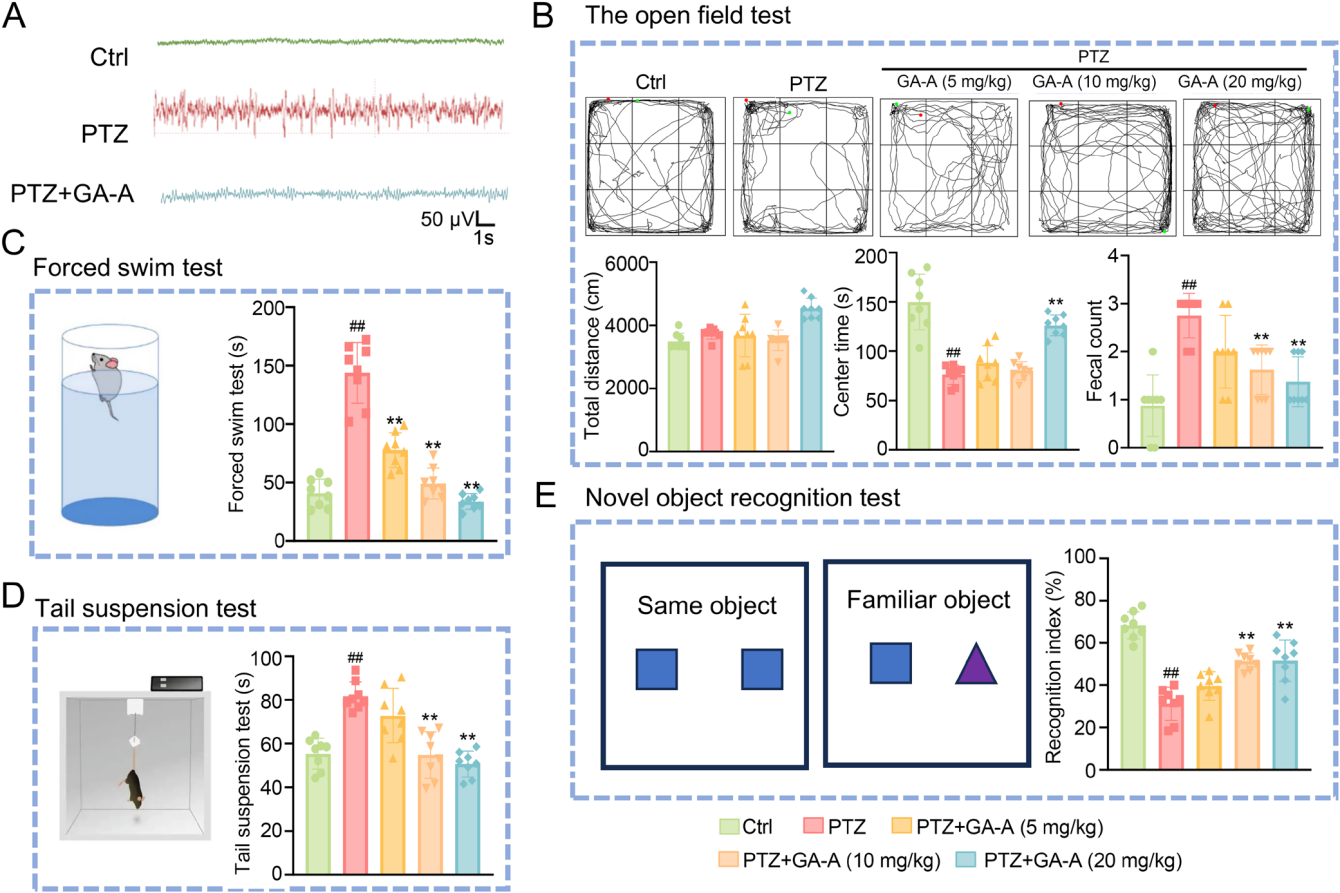
GA‐A alleviates PTZ‐induced epileptic seizures and improves anxiety‐ and depression‐like behavior. (A) Representative EEG recording. (B) The open field test. (C) The forced swim test. (D) The tail suspension test. (E) The novel object recognition test. Data are presented as mean ± SD (*n* = 8 per group). ^##^ *p* < 0.01 compared with the Ctrl group; **p < 0.01 compared with the PTZ group.

Representative EEG traces showing seizure activity in Ctrl, PTZ‐treated, and PTZ + GA‐A‐treated mice. PTZ administration induced prominent epileptiform discharges, which were markedly attenuated by GA‐A treatment (Figure 3A). In the OFT, no significant differences were observed among all groups, indicating intact general locomotor activity. PTZ‐treated mice spent significantly less time in the central zone compared with the Ctrl mice, reflecting increased anxiety‐like behavior. GA‐A treatment dose‐dependently restored center time, with a significant improvement observed at 20 mg/kg (Figure 3B). In the forced swim test, PTZ‐treated mice showed a significant increase in immobility time compared with the Ctrl mice, indicative of depressive‐like behavior. All GA‐A doses (5, 10, and 20 mg/kg) significantly reduced immobility time, demonstrating antidepressant‐like effects (Figure 3C). Consistent with FST results, in the tail suspension test, PTZ mice displayed prolonged immobility time, which was significantly reversed by GA‐A treatment at all doses (Figure 3D). In the novel object recognition test, the recognition index was significantly lower in the PTZ mice compared with the Ctrl mice, indicating cognitive impairment. GA‐A treatment dose‐dependently increased the recognition index, with significant effects observed at 10 and 20 mg/kg, suggesting improved recognition memory (Figure 3E). Thus, the above results indicated that GA‐A treatment alleviates PTZ‐induced epileptic seizures and improves anxiety‐ and depression‐like behavior.

### 3.4 Activating KCNQ2 May Be One of the Targets of GA‐A in Alleviating Epilepsy

**FIGURE 4:**
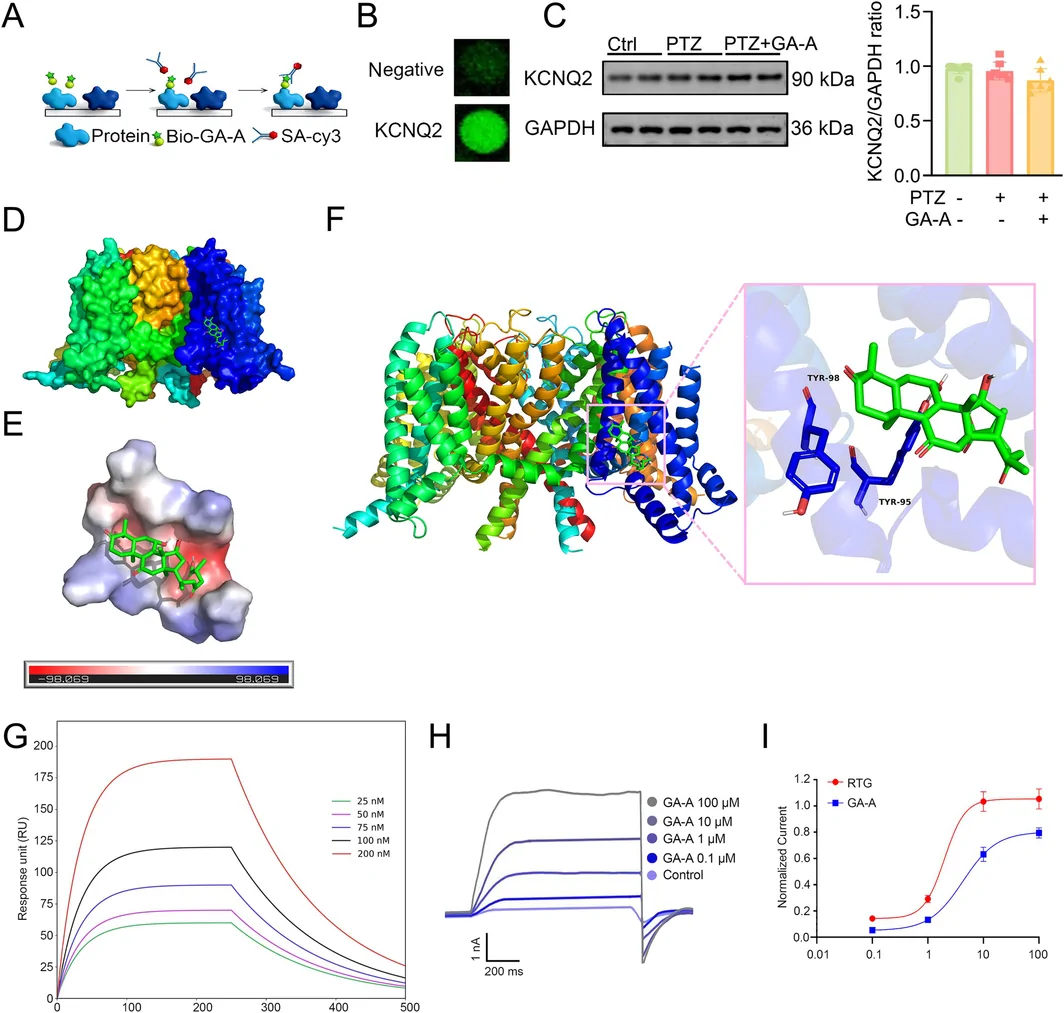
Activating KCNQ2 may be one of the targets of GA‐A in alleviating epilepsy. (A) Protein chip action mode diagram. (B) Representative images of KCNQ2 combination. (C) Representative Western blot bands of KCNQ2 protein expression in mouse hippocampal tissues. Data are presented as mean ± SD (*n* = 6 per group). (D, E) Prediction of docking between GA‐A and KCNQ2. (D) Electronic Cloud Density Map. (E) GA‐A is located within the cavity of the KCNQ2 protein. (F) Detailed interactions of GA‐A within the protein cavity, the carboxyl groups of GA‐A form 2 hydrogen bonds with the KCNQ2 protein, respectively. (G) SPR analysis of the binding affinity between GA‐A and the KCNQ2 protein. Serially diluted GA‐A solutions were injected onto the surface of the immobilized KCNQ2 chip. KD was calculated from 3 independent experiments with a global fitting algorithm. (H) Whole‐cell patch clamp. (I) GA‐A activates KCNQ2 to generate M current. Data are presented as mean ± SD (*n* = 6 per group).

To investigate the underlying mechanism of GA‐A in alleviating epilepsy, HuProT human protein assay was employed to capture direct protein interactors of GA‐A, followed by streptavidin‐Cy3 staining for visualization (Figure 4A). We identified 199 proteins that interact with GA‐A. Specific binding of GA‐A to KCNQ2 was observed, with strong green fluorescence (SNR: 36.90, log_2_ SNR: 5.21, Figure 4B). This directly identifies KCNQ2 as a GA‐A‐binding target. Then, KCNQ2 protein expression in the brains of the mice was detected. As shown in Figure 4C, by using Western blot, we found that PTZ injection and GA‐A treatment showed no remarkable changes in KCNQ2 protein expression. Therefore, we speculate that GA‐A may improve epilepsy by binding to KCNQ2 and affecting its channel activity.

Using molecular docking, the binding of GA‐A and KCNQ2 was predicted. The crystal structure of KCNQ2/3 (PDB ID: 7CR0), which contains A‐D chain, was adopted, and both KCNQ2 and KCNQ3 subunits were selected for all docking calculations because they exhibit complete structural integrity and no missing key amino acid residues in the ligand‐binding domain, which ensures the accuracy and reliability of the docking simulation results. Moreover, the binding energy is −6.896 kcal/mol. The results show that the carboxyl groups on GA‐A form two hydrogen bonds with amino acids: TYR‐98 and TYR‐95 of KCNQ2. GA‐A may insert into the hydrophobic pocket between the S3 and S4 segments of the KCNQ2/3 channel voltage‐sensing domain like a wedge (Figure 4D–F). We speculate that GA‐A activates KCNQ2/3 by stabilizing the activation conformation of S4 and keeping the voltage‐sensing domain in an activated state. In addition, we also compared the binding pocket of GA‐A with the binding pocket of the traditional KCNQ2/3 activator RTG (PDB: 7CR2). The results show that the sites and patterns at which RTG and GA‐A act are different. Bulk densities arising from the RTG molecule are observed in a hydrophobic pocket formed by S5, pore helix, and S6 from the neighboring subunit at the inter‐subunit interface in the pore domain. RTG binds to KCNQ2 mainly by hydrogen bonds with the side chain of Trp236, Ser303, and the main chain carbonyls of Leu299, Phe305, as well as hydrophobic interactions with residues Trp236, Phe240, Leu243, Leu272, Leu299, Phe304, and Phe305 [28].

In the above experiments, we confirmed that KCNQ2 is a potential target for GA‐A in the treatment of epilepsy. SPR shows that GA‐A binds to KCNQ2 in a dose‐dependent manner, with a KD of 68 nM (Figure 4G). Whole‐cell patch‐clamp recordings revealed that GA‐A, at concentrations ranging from 0.1 to 100 μM, activated Kv7.2/Kv7.3 channels in a concentration‐dependent manner (Figure 4H), with a half‐maximal effective concentration of 1.99 μM for RTG and 4.284 μM for GA‐A (Figure 4I).

### 3.5 *Kcnq2* Knock‐Out Did Not Affect the Alleviating Effect of GA‐A on Neuroinflammation and Oxidative Stress

**FIGURE 5:**
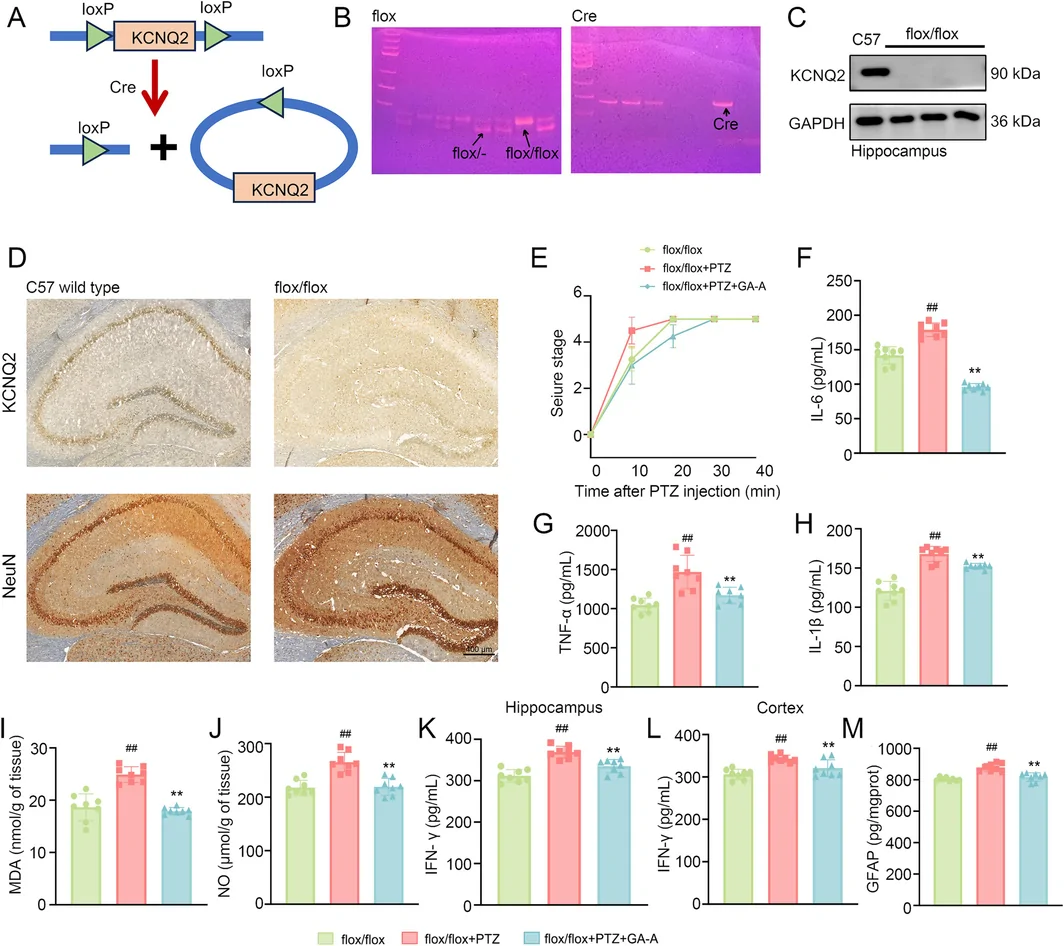
KCNQ2 knockout did not affect the alleviating effect of GA‐A on neuroinflammation and oxidative stress. (A) Hippocampal‐specific KCNQ2 knockout pattern diagram. (B) Representative gene identification map. (C) The expression level of KCNQ2 in hippocampal tissue. (D) The expression levels of KCNQ2 and NeuN in hippocampal tissue, scale bar = 400 μm. (E) The time‐course analysis of seizure progression. Data were shown as mean ± SD, *n* = 8. (F) Serum IL‐6 level. (G) Serum TNF‐α level. (H) Serum IL‐1β level. (I) Hippocampus MDA level. (J) Hippocampus NO level. (K) Hippocampus IFN‐γ level. (L) Cortex IFN‐γ level. (M) Hippocampus GFAP concentration. Data were shown as mean ± SD, *n* = 8, ^##^ *p* < 0.01 compared with the Ctrl group; **p < 0.01 compared with the PTZ group.

A Cre‐loxP system was employed to establish hippocampal‐specific *Kcnq2* knockout mice (Figure 5A). The representative gene identification gel diagram is shown in Figure 5B. Protein was extracted from mouse hippocampal tissue for Western blot. The results showed that KCNQ2 expression was detected in the hippocampal tissue of wild‐type C57 mice, while no KCNQ2 protein expression was detected in hippocampal‐specific *Kcnq2* knockout (flox/flox) mice (Figure 5C). The results indicate successful hippocampal‐specific knockout of *Kcnq2*. Immunohistochemistry showed that KCNQ2 expression was distributed in the hippocampus of C57 wild‐type mice, while it was not expressed in flox/flox mice. Moreover, knockout of *Kcnq2* did not affect the expression and distribution of NeuN (Figure 5D).

Next, we established a PTZ‐induced mouse epilepsy model in flox/flox mice. Administration of PTZ resulted in the high seizure stage of epilepsy, while administration of GA‐A prolonged the time for seizures to reach level four after PTZ administration, but did not reduce the severity of seizures (Figure 5E). Subsequently, we detected the expression of pro‐inflammatory cytokines in the serum of each group of mice. After PTZ treatment, the expression levels of serum IL‐6 (Figure 5F), TNF‐α (Figure 5G), and IL‐1β (Figure 5H) were significantly increased, and there was a significant inhibitory effect after GA‐A. The levels of oxidative stress products MDA and NO also significantly increased after PTZ administration, and GA‐A had a significant inhibitory effect on them (Figure 5I,J). In the hippocampus and cortex tissues of mice, after administration of GA‐A, the significantly increased IFN‐γ in the model group was significantly inhibited (Figure 5K,L). PTZ administration also significantly increased GFAP expression in mouse hippocampal tissue, which slightly decreased after GA‐A administration, with statistically significant differences compared to the PTZ group (Figure 5M). The above results indicate that hippocampal‐specific *Kcnq2* knockout does not seem to affect the inhibitory effect of GA‐A on PTZ‐induced neuroinflammation and oxidative stress.

### 3.6 *Kcnq2* Knock‐Out Weakened the Improvement Effect of GA‐A on EEG and Anxiety‐ and Depression‐Like Behavior

**FIGURE 6:**
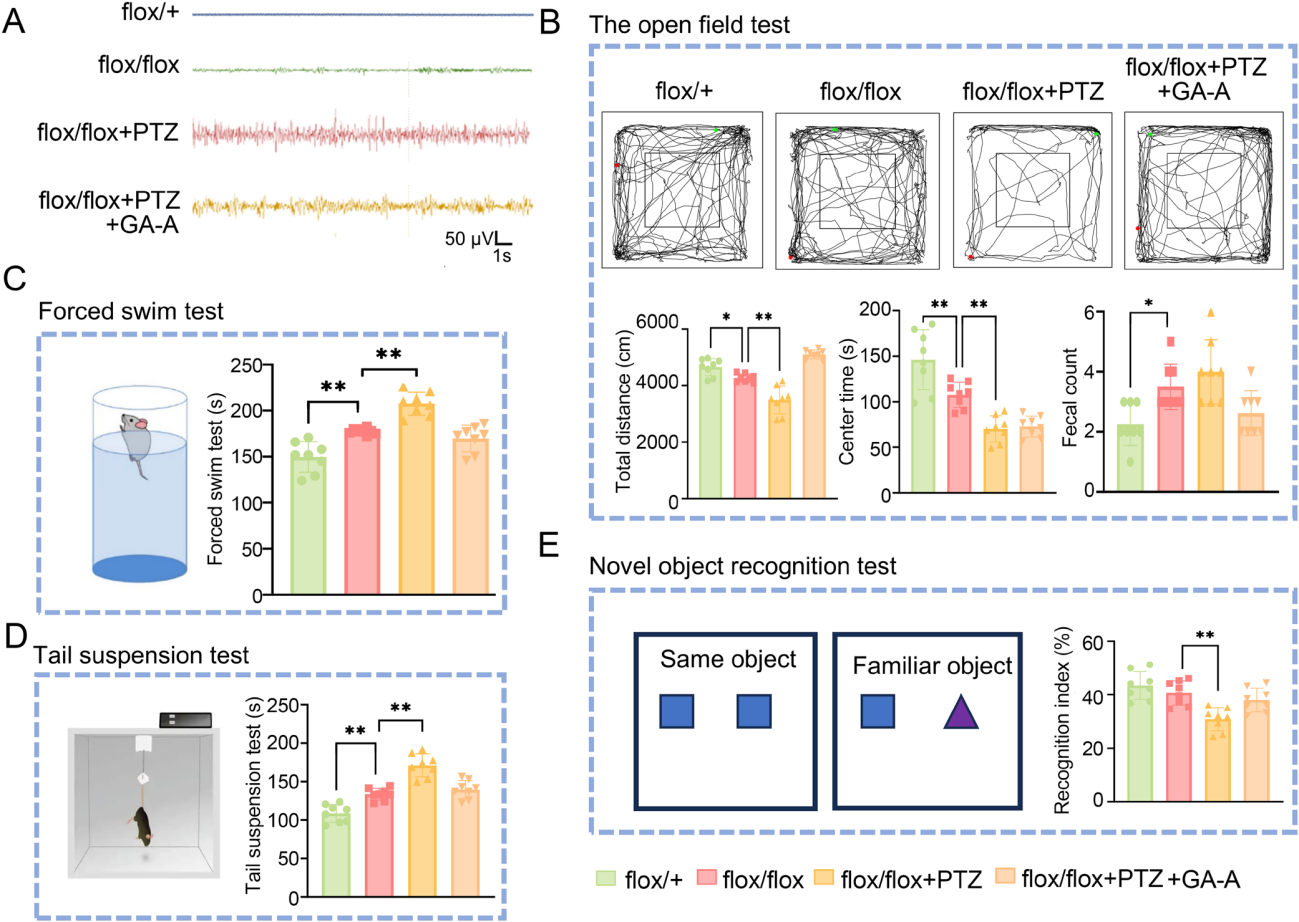
*Kcnq2* knockout weakened the improvement effect of GA‐A on EEG and anxiety and depression like behavior. (A) Representative EEG images of Hippocampal‐specific *Kcnq2* Knockout mice. (B) The open field test in hippocampal‐specific *Kcnq2* knockout mice. (C) The forced swim test in hippocampal‐specific *Kcnq2* knockout mice. (D) The tail suspension test in hippocampal‐specific *Kcnq2* knockout mice. (E) The novel object recognition test in hippocampal‐specific *Kcnq2* knockout mice. Data are presented as mean ± SD, *n* = 8. *p < 0.05, **p < 0.01 compared with the corresponding group.

Similarly, we also recorded the EEGs of *Kcnq2* hippocampal‐specific knockout mice in a conscious state. The EEG activity of the flox/flox group mice was stable with low amplitude, although slightly chaotic compared to flox/+ mice; however, it was still in a normal baseline state. After PTZ administration, a large number of high‐amplitude and high‐frequency epileptic discharges appeared in flox/flox mice, which are typical of an epileptic seizure EEG. The amplitude and frequency of epileptic discharges in GA‐A‐treated mice were significantly reduced, although there was no significant improvement compared to that in wild‐type mice treated with GA‐A (Figure 6A). The results showed that hippocampal‐specific knockout of *Kcnq2* weakened the antiepileptic effect of GA‐A.

In the OFT, PTZ‐treated mice were unwilling to explore the central region and spent less time in the center compared to flox/flox mice, despite walking similar distances between the two groups of mice (Figure 6B). The reduction in central region time is associated with higher levels of anxiety‐ and depression‐like behavior, indicating that KCNQ2 deficiency exacerbates these behaviors in epileptic mice. The FST (Figure 6C) and TST (Figure 6D) showed a significant prolongation of immobility time and a significant decrease in recognition index in flox/flox mice, indicating an exacerbation of depressive‐like despair behavior and significant impairment of cognitive and memory function. There was no significant change in the index, indicating a significant impairment of cognitive and memory function, which was not improved after administration of GA‐A (Figure 6E). These results indicate that GA‐A does not significantly improve anxiety‐ and depression‐like behavior in *Kcnq2*‐knockout epileptic mice.

## 4 Discussion

In this study, we established a PTZ‐induced mouse model in wild‐type and hippocampal‐specific *Kcnq2* knockout mice and investigated the potential of GA‐A in alleviating epilepsy by inhibiting neuroinflammation, reducing neuronal apoptosis, activating the KCNQ2 channel, and evaluating behavioral alterations. The present study revealed that activation of KCNQ2 represents a key target for GA‐A to ameliorate epileptiform electroencephalogram abnormalities and attenuate epilepsy‐associated anxiety‐ and depression‐like behaviors. However, the activation of this target is largely independent of the inhibitory effects of GA‐A on neuroinflammation and oxidative stress. GA‐A may ameliorate epilepsy through multiple pathways and targets, suggesting that GA‐A could serve as a candidate lead compound for the treatment of epilepsy.

GA‐A is a triterpenoid compound isolated from *G. lucidum* . Previous studies have demonstrated that GA‐A exerts anti‐inflammatory and antioxidant effects and is beneficial against multiple diseases [29]. Recently, several studies have indicated that GA‐A can cross the blood–brain barrier and ameliorate several central nervous system diseases, such as Alzheimer's disease [30], Parkinson's syndrome [31], and cerebral ischemia [23]. It has also been reported that GA‐A inhibits lipopolysaccharide‐induced microglial inflammation in mice [19] and alleviates epilepsy by reducing cortical neuronal apoptosis via suppression of the mitogen‐activated protein kinase signaling pathway [18]. Although the antiepileptic effect of GA‐A has been documented, its therapeutic targets and underlying molecular mechanisms remain to be further elucidated.

In the present study, we investigated the protective effects of GA‐A against neuroinflammation and oxidative damage in a mouse PTZ model, which is consistent with previous studies [31, 32, 33]. Furthermore, by examining electroencephalograms in conscious mice and behavioral alterations, the present study confirmed that GA‐A ameliorates abnormal brain discharges and improves anxiety‐ and depression‐like behaviors in mice. Similarly, GA‐A has been shown to exert ameliorative effects on depression‐like behaviors in the lipopolysaccharide‐induced mouse depression model [31], chronic social defeat stress model [32, 34], and post‐stroke depression model [35].

Subsequently, KCNQ2 was verified as a direct target of GA‐A using protein microarray, molecular docking, and surface plasmon resonance assays. Kv7 channels represent a subtype of voltage‐gated potassium channels. The Kv7 channel family comprises five members, namely Kv7.1 to Kv7.5, which are encoded by the *KCNQ1* to *KCNQ5* genes, respectively. *KCNQ1* is predominantly expressed in cardiomyocytes, whereas *Kcnq2* to *Kcnq5* are mainly distributed in the nervous system and constitute the structural components of the M‐channel that regulates neuronal excitability. Neuronal M‐channels are mainly assembled as heterotetramers composed of Kv7.2/KCNQ2 and Kv7.3/KCNQ3 subunits. The M‐channel tetramer exhibits a typical domain‐swapping conformation, with a central pore domain surrounded by voltage‐sensing domains. This is the first time that KCNQ2 has been identified as one of the targets of GA‐A in alleviating epilepsy.

KCNQ2/3 channels are predominantly expressed in brain regions closely associated with epileptogenesis, including the cerebral cortex, hippocampus, thalamic areas, as well as the spinal dorsal root ganglia, spinal dorsal horn, and trigeminal ganglia involved in pain transmission pathways [36, 37]. These channels represent important therapeutic targets for various neurological disorders. Aberrant activity of KCNQ2/3 channels has been linked to severe pathological conditions. For instance, mutations in *Kcnq2* or *Kcnq3* cause benign familial neonatal seizures, and mutations in *Kcnq2* also lead to the development of epileptic encephalopathy [38, 39]. In our study, compared to wild‐type mice, *Kcnq2* hippocampal‐specific knockout mice showed relatively less stable brainwaves and increased anxiety‐and depression‐like behaviors, although there was no significant difference compared to the wild‐type mice, which may be due to individual differences in the animals. Changes in KCNQ2/3 channel activity have also been implicated in cerebral ischemia injury [40], age‐related memory impairment [41], neuroendocrine systems [42], alcohol addiction [41], and neural differentiation [43]. The mechanism of action of KCNQ2 in other diseases still needs further investigation.

In our study, following tissue‐specific knockout of *Kcnq2* in mice using the Cre‐loxP system, hippocampal *Kcnq2* knockout resulted in slightly disorganized but still within‐normal‐range brain EEGs. After PTZ administration, the severity of epilepsy was significantly higher, and the therapeutic efficacy of GA‐A was compromised, indicating that GA‐A alleviates epilepsy in a KCNQ2‐dependent manner. In *Kcnq2* knockout mice, the inhibitory effects of GA‐A on neuroinflammation and oxidative stress remained unaffected, suggesting that GA‐A ameliorates epilepsy through both KCNQ2‐dependent and ‐independent mechanisms.

Activation of neuronal Kv7 channels has emerged as an important strategy for epilepsy therapy owing to their role in regulating neuronal excitability [44]. RTG, an activator of Kv7.2/7.3 channels, was once approved for the treatment of epilepsy but was withdrawn in 2017 due to its adverse effects, including ophthalmological and skin pigmentation [45]. Despite these setbacks, Kv7.2/7.3 channels remain a promising target for the development of antiepileptic drugs. Previous studies have attributed the quinone/aminoquinone diamine metabolites of RTG and the associated blue discoloration to its electron‐rich triamine aromatic scaffold [45]. However, such a structural moiety is absent in GA‐A, and long‐term administration of GA‐A has been proven to be safe in previous studies [7]. Patch‐clamp experiments have also confirmed that GA‐A exerts effects similar to those of RTG and can activate Kv7 channels to generate M‐current.

However, this study has several limitations, including a relatively small sample size; the PTZ‐induced acute seizure model cannot fully recapitulate the complexity of human epilepsy; and the direct target engagement of GA‐A at KCNQ2 in vivo was not conclusively verified, thereby limiting the causal interpretation of our results. This requires more detailed and systematic research. In all, the Kv7 channel activation property of GA‐A makes it a promising candidate drug for the treatment of focal or systemic epilepsy.

## Author Contributions

**Yang Yao:** writing – original draft, investigation, formal analysis, visualization, funding acquisition, project administration. **Zhujun Liu:** investigation, formal analysis, validation. **Xujiao Wang:** methodology, investigation, formal analysis. **Cunhao Bian:** methodology, investigation, visualization. **Rui Wang:** methodology, writing – review and editing, supervision. **Yukun Zhang:** conceptualization, methodology, writing – review and editing, supervision, funding acquisition, project administration.

## Funding

This study was supported by the National Natural Science Foundation of China (82304493), the Major project of Chongqing Municipal Education Commission (KJZ‐M202302701), General project of Chongqing Science and Technology Bureau (CSTB2024NSCQ‐MSX0831), and the Youth Project of Chongqing Municipal Education Commission (KJQN202302701).

## Ethics Statement

For the animal experiments: All procedures were carried out in strict accordance with the Guidelines for the Care and Use of Laboratory Animals. Ethical approval was granted by the Animal Research Ethical Committee of the Chongqing Three Gorges Medical College (Ethical Approval Number: SXYZ‐A‐2306‐0011).

## Conflicts of Interest

The authors declare no conflicts of interest.

## Data Availability

The data that support the findings of this study are available from the corresponding author upon reasonable request.
